# Effect of glyphosate, its metabolite AMPA, and the glyphosate formulation Roundup^®^ on brown trout (*Salmo trutta* f. *fario*) gut microbiome diversity

**DOI:** 10.3389/fmicb.2023.1271983

**Published:** 2024-01-15

**Authors:** N. Hembach, V. Drechsel, M. Sobol, A.-K. Kaster, H.-R. Köhler, R. Triebskorn, T. Schwartz

**Affiliations:** ^1^Karlsruhe Institute of Technology (KIT), Institute of Functional Interfaces, Karlsruhe, Germany; ^2^Institute of Evolution and Ecology, Animal Physiological Ecology, University of Tübingen, Tübingen, Germany; ^3^Karlsruhe Institute of Technology (KIT), Institute for Biological Interfaces, Karlsruhe, Germany

**Keywords:** glyphosate, Roundup^®^, AMPA, fish microbiome, gut diversity, *Salmo trutta* f. *fario*

## Abstract

Glyphosate is used worldwide as a compound of pesticides and is detectable in many environmental compartments. It enters water bodies primarily through drift from agricultural areas so that aquatic organisms are exposed to this chemical, especially after rain events. Glyphosate is advertised and sold as a highly specific herbicide, which interacts with the EPSP synthase, an enzyme of the shikimate metabolism, resulting in inhibition of the synthesis of vital aromatic amino acids. However, not only plants but also bacteria can possess this enzyme so that influences of glyphosate on the microbiomes of exposed organisms cannot be excluded. Those influences may result in subtle and long-term effects, e.g., disturbance of the symbiotic interactions of bionts with microorganisms of their microbiomes. Mechanisms how the transformation product aminomethylphosphonic acid (AMPA) of glyphosate might interfere in this context have not understood so far. In the present study, molecular biological fingerprinting methods showed concentration-dependent effects of glyphosate and AMPA on fish microbiomes. In addition, age-dependent differences in the composition of the microbiomes regarding abundance and diversity were detected. Furthermore, the effect of exposure to glyphosate and AMPA was investigated for several fish pathogens of gut microbiomes in terms of their gene expression of virulence factors associated with pathogenicity. *In vitro* transcriptome analysis with the fish pathogen *Yersinia ruckeri* revealed that it is questionable whether the observed effect on the microbiome is caused by the intended mode of action of glyphosate, such as the inhibition of EPSP synthase activity.

## 1 Introduction

The importance of trace substances for the integrity of aquatic ecosystems has been widely discussed for years, not only in the scientific community but also among the general public and in policy. Of particular, interest is whether there is a causal link between the presence of chemicals in water bodies and the ongoing biodiversity crisis, which manifests itself in the decline of species and changes in the diversity and structure of biocenoses (Baranov et al., 2020). According to the Living Planet Index, one can assume an annual decline of 3.9% in the populations of freshwater organisms (Beaufort et al., 2017). The integrity of fish populations, in particular, has deteriorated significantly in recent years (LUBW, 2015). To be able to stop or counteract these negative developments, causes and drivers must be known and their mode of action has to be better understood. Usually, in this context, the mentioned reasons are increasing habitat loss, climate change, the spread of invasive species but also chemical inputs, whereby the interaction of several factors is likely (Sigmund et al., 2023).

Chemicals occur in surface waters in large numbers and as trace substances. They represent a chronic burden for organisms. As high glyphosate concentrations are rare in environmental waters in industrial countries, acute effects of glyphosate or AMPA on aquatic organisms are not often reported. Of particular, importance in this context is that many chemicals, such as pharmaceuticals or pesticides, have very specific mechanisms of action or cause harm to natural populations indirectly via the interplay of effect cascades so that their effects are not detected by the usual test systems used for risk assessment (Köhler and Triebskorn, 2013). This is particularly problematic when substances cause sub-organismic, subtle, and long-term effects, e.g., by modulating immunocompetence, thereby reducing the fitness and resilience of organisms (Segner et al., 2011; Rehberger et al., 2017).

In recent years, the gut microbiome has become increasingly important as a target for substance effects (Wu and Wu, 2012; Rooks and Garrett, 2016). According to the *holobiont principle*, which has recently received increasing attention in life sciences (Bosch and Miller, 2016), individual health depends on an intact symbiosis of the gut microbiome. Here, the term “holobiont” was coined to describe the entity of a host and its microbiome. In mammals, there is increasing evidence that gut dysbiosis can lead to obesity, diabetes, or other chronic diseases, which have increased their frequency during the last decades (Bosch and Miller, 2016; Deriu et al., 2016; Berer et al., 2017). It has also been shown that certain pesticides, pharmaceuticals, or artificial sweeteners can alter gut microbiome composition (Cox and Blaser, 2013; Samsel and Seneff, 2013; Bokulich and Blaser, 2014; Pollak, 2017). Moreover, it is known that, conversely, microorganisms in the gut can also modulate the properties and toxicity of chemicals (Claus et al., 2016).

Pesticides reaching aquatic ecosystems can affect organisms either directly or indirectly, in the latter way, e.g., by interacting with symbionts such as gut bacteria. In vertebrates, the gut microbiome and its implications for the immune status and health of the host organism have become the center of various studies in recent years, even though causal relationships are just the beginning of being understood. Despite its controversially discussed use in agriculture, only few publications focus on the effect of glyphosate on the host microbiome and the therein incorporated health risk for the host (Motta et al., 2018).

Glyphosate is used worldwide and is detectable in many environmental compartments in increasing concentrations. It is introduced into water bodies through run-off from agricultural land. Environmental concentrations range from 0.0001 to 105 g mL^−1^ (Lanzarin et al., 2023). Up to 164 μg L^−1^ of glyphosate can be found in surface water in European countries (Busch and Reupert, 2013; LAWA – Bund/Länder-Arbeitsgemeinschaft Wasser, 2016; Carles et al., 2019; Tauchnitz et al., 2020). After accidents, concentrations in water can even reach 1.7–5.2 mg L^−1^ (Glozier et al., 2012). Up to 150 μg kg^−1^ dry weight of glyphosate has been found in freshwater sediments (Widenfalk et al., 2008). Aquatic biota, including fish, are thus exposed to the highly water-soluble herbicide via the water and sediment phases. The herbicide can bind to organic material such as humic acids and also can be taken up into cells via amino acid transporters (Piccolo et al., 1996; Xu et al., 2016a). Glyphosate can thus be expected to enter the intestinal lumen of organisms through food and reach their tissues via membrane passage using amino acid transporters (Xu et al., 2016b). From a chemical point of view, glyphosate is a derivative of the amino acid glycine, which is a part of most proteins in living systems. It was designed to interfere with the shikimate pathway, preventing the biosynthesis of aromatic amino acids (phenylalanine, tyrosine, and tryptophan) (Schönbrunn et al., 2001). All plants possess this metabolic pathway. Animal organisms themselves lack it, which is why glyphosate producers claim that side effects in animals are highly unlikely and implausible. However, this does not consider fungi and many microorganisms, including those that colonize animal hosts and are relevant for host health, can also possess the shikimate metabolism.

This study therefore focuses on the influence of the herbicide glyphosate on the gut microbiome of brown trout (*Salmo trutta* f*. fario*). The brown trout was chosen as the object of the study due to its ecological relevance in freshwater ecosystems in view of repeatedly reported declines in field populations (Burkhardt-Holm and Scheurer, 2007; Kuehn et al., 2018). The causes of population declines are often unknown, or multifactorial reasons are quoted. Kennedy (2018) considers it likely that the decline in Pacific salmonid populations may be related to glyphosate-based herbicides, although this herbicide is described by the manufacturer, which is very specific to plants (Franz et al., 1997; Duke and Powles, 2008; Blake and Palett, 2018). In the present project, we test the toxicity of the herbicide to the fish microbiome and attempt to determine whether the mechanism for any toxicity observed is via inhibition of the shikimate pathway. For this purpose, we exposed juvenile brown trout (*Salmo tutta* f. *fario*) to different concentrations of glyphosate, one of its formulations (Roundup^®^ LB Plus), and its main metabolite aminomethylphosphonic acid (AMPA) and investigated the impact on the gut microbiome. In parallel, studies were conducted on the health status of identical fish by means of histological, biochemical, and molecular biological analyses. These results, however, are not presented in the present study; otherwise, the scope of this article would be exceeded. This study thus exclusively contains data on shifts in the composition of the microbiome with a comprehensive view on population densities and diversities. We particularly focused on fish pathogens and their virulence gene expression, which is relevant to the health status of the host fish.

## 2 Materials and methods

### 2.1 Experimental procedure

The test substances were glyphosate [N-(phosphonomethyl) glycine; Sigma–Aldrich, Merck KGaA, Darmstadt, Germany], the formulation Roundup^®^ LB Plus (purchased at a local retail store), and the metabolite AMPA [(aminomethyl)phosponic acid; Acros Organics BVBA, Geel, Belgium]. These compounds were dissolved in ultrapure water to obtain solutions of 56, 560, and 5,600 μg L^−1^ for glyphosate and Roundup^®^ (containing 560 or 5,600 μg L^−1^ gLyphosate, respectively) and 3,666 μg L^−1^ for AMPA. To achieve the respective nominal concentrations, corresponding stock solutions were prepared with ultrapure water.

Brown trout (*Salmo trutta* f. *fario*) was purchased from a commercial trout farm in southern Germany (Forellenzucht Lohmühle, Alpirsbach-Ehlenbogen, Germany), which is listed as disease-free according to the EC Council Directive (2006). The fish were acclimated to laboratory conditions in a climate chamber in a 200 L tank with filtered (iron filter, activated charcoal filter, and particle filter) and aerated tap water 1 week prior to the exposure experiment. The fish were observed daily and fed with commercial trout feed (Inico Plus, Biomar, Brande, Denmark). All animal experiments were approved by the Animal Welfare Committee of the Regional Council of Tuebingen, Germany (approval number ZO 2/16 and ZO 02/21 G).

Each exposure experiment was conducted in a semi-static, three-block setup. Therefore, each treatment was set up in triplicates, and each block consisted of six aquaria tanks containing 15 L of the respective test medium. In each aquarium tank, 30 fish of 6 months old or 10 fish of 10 months old were exposed to water with no test medium as control, or to 56 μg L^−1^, 560 μg L^−1^, or 5,600 μg L^−1^ of glyphosate, Roundup^®^ LB Plus containing 560 μg L^−1^ or 5,600 μg L^−1^ of glyphosate, respectively, or 3,666 μg L^−1^ AMPA, which is equimolar to the highest concentration of glyphosate. Exposure experiments were conducted under a 12:12 light/dark cycle at 7°C, and the tanks were covered with black foil to protect the fish from direct light. Half of the respective test solutions were removed from each tank every 2–3 days and replaced with fresh test solutions to keep the concentration stable. Furthermore, water conditions (temperature, conductivity, pH, and oxygen content) were checked regularly. The fish were exposed for 3 weeks and then anesthetized and killed by an overdose of the anesthetic MS-222 (tricaine methanesulfonate; Pharmaq, Overhalla, Norway), followed by a cervical cut. Various samples were collected for different biomarkers including gill and gut samples for microbiome analysis.

### 2.2 Sample preparation

For population analysis and the abundance of fish pathogens, 90 guts of 6-month-old fish and 90 guts of 10-month-old juvenile fish were utilized to extract bacterial DNA. For bacterial DNA extraction, the midgut part was used. Due to the small tissue size, no separation of mucus-associated biofilm and fish tissue was performed. Instead, the whole gut was dissolved by a 3-h incubation using lysing buffer and proteinase K provided by DNA Mini Kit by QIAGEN (Hilden, Germany). The DNA extraction and clean-up were performed by the DNA Mini Kit. The number of intestines analyzed individually ranged from 8 to 18 in biological triplicates and technical replicates, depending on the exposure and age of the fish.

### 2.3 *In vivo* microbiome analysis by terminal restriction fragment length polymorphism (t-RFLP)

Terminal Restriction Fragment Length Polymorphism (t-RFLP) was applied to assess population changes in the microbiome of 6-month and 10-month-old fish larvae. In principle, t-RFLP relies on the universal amplification of a large 16S rRNA fragment from *Eubacteria*. Using selected restriction enzymes, it is possible to generate labeled, terminal 16S rRNA fragments of different sizes depending on individual restriction sites of the present bacterial species. As a consequence, this fingerprint analysis complements and extends the results of amplicon sequences, aiming on possible shifts in the microbial population. The t-RFLP is not designed and qualified to detect specific species but specified terminal fragments of the 16S rRNA which can correlate with closely affiliated bacteria. To compensate for these uncertainties, the different fragments representing different species are called phylogenetic clusters.

Sample preparation for t-RFLP analysis was performed in technical duplicates. For the DNA-based t-RFLP analysis, a universal PCR was performed using a Fam-labeled primer system targeting the V1-V3 region of the 16S rRNA. The primer sequences utilized are presented in Table 1. The mastermix for each PCR reaction consisted of 19 μL nuclease-free water, 2.5 μL combination buffer, 0.5 μL dNTP (VWR, Darmstadt, Germany), 0.125 μL Hot Start Polymerase (VWR, Darmstadt, Germany), and 1 μl of each primer (20 nM) for a final reaction volume of 25 μL. The PCR profile included 15 min of initial denaturation at 95°C, 30 s of denaturation at 95°C, 30 s of annealing at 56°C, 60 s of cycle elongation at 72°C, and 10 min of final elongation at 72°C for 30 cycles. Afterward, the PCR product was restricted using the four-base cutting Fast Digest Enzyme *Hha*I (Thermo Fisher Scientific, Waltham, United States). In total, 10 U was used to restrict 0.2 μg of PCR product at 37°C for 3 h. After restriction, the technical duplicates were pooled again, and 2 μL of the restricted DNA was mixed with 15 μL of Hi-Di formamide; fragment analysis was performed at a SeqStudio device (Thermo Fisher Scientific, Waltham, United States) to detect the FAM-labeled terminal restriction fragment. The run utilized an injection time of 40 s at 1200 Volts and a run time of 1,440 s at 9000 Volts. Analysis range of the t-RFLP was set to 20–500 bp with a set bin width of 5 bp. All samples were analyzed within one run, and the normalization was performed over the sum of signals. The resulting fragmentation patterns were further analyzed using R and the vegan community ecology package (Oksanen et al., 2022). Calculations were performed using the respective peak areas.

**Table 1:** Utilized primers, their sequences targeting fish pathogen virulence factors.

Target	Positive control	Primer sequence		Size	Reference
PCR 16S rDNA		F	TCCTACGGGAGGCAGCAGT	195	Muyzer et al. (1993)
		R	ATTACCGCGGCTGCTGG		
t-RFLP 16S rDNA		F	AGAGTTTGATCCTGGCTCAG*-FAM	490	
		R	ATTACCGCGGCTGCTGG		
aerA (*A. salmonicida*)	*Aeromonas salmonicida* DSM 19634	F	CAAGAACAAGTTCAAGTGGCCA	309	Stanley et al. (1994)
		R	ACGAAGGTGTGGTTCCAGT		
act (*A. salmonicida*)		F	GAGAAGGTGACCACCAAGAACA	232	Bin Kingombe et al. (2010)
		R	AACTGACATCGGCCTTGAACTC		
alt (*A. salmonicida*)		F	TGCTGGGCCTGCGTCTGGCGG	361	
		R	AGGAACTCGTTGACGAAGCAGG		
ast (*A. salmonicida*)		F	GCATCGAAGTCACTGGTGAAGC	536	
		R	CGGCGACTCAACGTTTGAC		
ace (*E. faecalis*)	*Enterococcus faecalis* DSM 20478	F	CGGCGACTCAACGTTTGAC	100	Rich et al. (1999)
		R	TCCAGCCAAATCGCCTACTT		
gelE (*E. faecalis*)		F	CGGAACATACTGCCGGTTTAGA	100	Qin et al. (2001)
		R	TGGATTAGATGCACCCGAAAT		
efaA (*E. faecalis*)		F	TGGGACAGACCCTCACGAATA	100	Lowe et al. (1995)
		R	CGCCTGTTTCTAAGTTCAAGCC		
esp (*E. faecalis*)		F	GGAACGCCTTGGTATGCTAAC	100	Shankar et al. (1999)
		R	GCCACTTTATCAGCCTGAACC		
esp (*E. faecium*)	*Enterococcus faecium* DSM 20477	F	CTTTCGACGTGGATGTAGAGTTTG	70	Rathnayake et al. (2012)
		R	GGTACGTATGTTGCATCATTTTCC		
gelE (*E. faecium*)		F	TCAGTGGTGTCAGCAGCCTTT	85	
		R	TGGTTTACCTGAATGTCTTCTTTAGC		
flgA (*Y. ruckeri*)	*Yersinia ruckeri* DSM 18506	F	GTGCCGCTGACAATCTGG	217	Huang et al. (2013)
		R	CCAAGGGAACTCTGGCTTTG		
rucC (*Y. ruckeri*)		F	CGAAAGGCTCCAACTGACTG	414	
		R	CAGAAGGCGGTGTTTTGCTC		
lnv (*Y. ruckeri*)		F	GTTACCGGTCTTACCTCAGTTAG	136	Wrobel et al. (2018)
		R	GAATGGTGTATAGGTTATCCCCG		
ilm (*Y. ruckeri*)		F	CGTCAATGAGGACTTCCATCT	124	
		R	GAGGGTATTACGGCTGTCTTT		
gyr (*Y. ruckeri*)		F	ACCAGTAGCCGATCAATAAAGTC	142	
		R	AGGAAGATGTGGTTGTGACG		
*A. salmonicida*	*Aeromonas salmonicida* DSM 19634	F	CGGAACGTAATCTGAATTGTTCTTTTC	131	Balcázar et al. (2007)
		R	ATTGCTTATCGAGGCAGCCAAC		
*Y. ruckeri*	*Yersinia ruckeri* DSM 18506	F	GCGAGGAGGAAGGGTTAAGTG	70	This study
		R	GTTAGCCGGTGCTTCTTCTG		
*F. psychrophilum*	*Flavobacterium psychrophilum* DSM 3660	F	GAGTTGGCATCAACACAC	146	Langevin et al. (2012)
		R	TCCGTGTCTCAGTACCAG		
*E. faecium*	*Enterococcus faecium* DSM 20477	F	GCAGCCACCAATTTACAACGA	56	Frahm and Obst (2003)
		R	TCATCTGCCAAATTCTCTGAGG		
*E. faecalis*	*Enterococcus faecalis* ATCC 51299	F	CACCTGAAGAAACAGGC	475	Depardieu et al. (2004)
		R	ATGGCTACTTCAATTTCACG		

Primers for 16S rDNA genes are used as housekeeping system for gene expression analyses. The asterisk-marked sequence is FAM labeled. F: forward; R: reverse; Size: amplicon size in base pairs.

### 2.4 *In vitro* analysis of virulence gene detection and expression in fish pathogens

For analysis of the virulence gene expression, a variety of different virulence factors of the observed fish pathogens were chosen (Table 2).

**Table 2:** Virulence genes associated with the different fish pathogen and their biological activities under investigation.

*Yersinia ruckeri*		*Enterococcus faecalis*		*Aeromonas salmonicida*		*Enterococcus faecium*	
flgA	Flagellar secretion apparatus	ace	Regulates collagen	aerA	Aerolysin	gelE	Regulates biofilm formation
Ilm	Invasin-like molecule	gelE	Regulates biofilm formation	act	Enterotoxin (aerolysin-related)	esp	Biofilm formation
Inv	Invasin	esp	Biofilm formation	alt	Heat-labile lipase		
rucC	Ruckeribactin	efaA	Cell adhesion	ast	Heat-stable lipase		

The reference bacteria *Y. ruckeri* (DSM 18506), *E. faecalis* (DSM 20478), *A. salmonicida* (DSM19634), and *E. faecium* (DSM 20477) were incubated overnight in full media at their respective temperature. After incubation overnight, 5 mL of the culture was pelleted, washed twice with PBS, and resuspended to an OD_600_ of 0.1 with a new medium containing 0 μg L^−1^, 1 μg L^−1^, 100 μg L^−1^, 560 μg L^−1^, and 5,600 μg L^−1^ glyphosate or AMPA and incubated. At the end of the exponential growth phase, the bacteria were pelleted again at 4°C, and the total RNA was extracted using the RNeasy Mini Kit (QIAGEN, Hilden), including additional DNase treatment, according to the manufacturer’s instructions. Reverse transcription was performed using random hexamers and the Superscript IV reverse transcriptase (Thermo Fisher Scientific, Waltham, United States), according to the manufacturer’s instructions. Quantification was performed by qPCR using the Maxima SYBR Green Master Mix (Thermo Fisher Scientific, Waltham, United States), utilizing the primers presented in Table 1. 16S rRNA expression was used as reference housekeeping gene for normalization (Huang et al., 2014; Bolhari et al., 2018). Analysis of all virulence factors was performed in biological triplicates.

### 2.5 Whole transcriptome analysis

The exposure of the reference bacteria *Y. ruckeri* (DSM18506) for the whole-transcriptome analyses was carried out in biological duplicates in the same way for the detection of virulence factors. After washing with PBS, the pelleted overnight culture was re-suspended in either full medium (tryptogenic soy broth) containing amino acids (VWR, Darmstadt, Germany) or mineral medium (M9 medium containing 6 g L^−1^ Na_2_HPO_4_. 3 g L^−1^ KH_2_PO_4_, 0.5 g L^−1^ NaCl, 1 g L^−1^ NH_4_Cl, 0.1 mL 1 M CaCl_2,_ 2 mL 1 M MgSO_4_, and 5 mL 40% glucose, adjusted to pH 7.4) without any amino acids. RNA isolation was performed using the RNeasy Mini Kit from QIAGEN (Hilden, Germany), including DNase digestion. Libraries were created using the ZymoResearch RiboFree Total RNA Library Kit (ZymoResearch, Freiburg, Germany) and subsequent electrophoretic quality control. An Illumina NextSeq 500/550 HighOutput Kit V2.5 with 400 million read pairs was used for sequencing. The quality control and the analysis of the sequencing were carried out using the following programs: FastQC (quality control), TrimGalore (removal of adapter sequences), SortMeRNA (removal of rRNA), STAR (alignment of sequences with gene databases), SAMtools (sorting and indexing of individual aligned sequences), FeatureCounts (counting the number of identical aligned sequences), and DESeq2 (normalization of count and expression analysis) (Kopylova et al., 2012; Liao et al., 2014; Love et al., 2014; Dobin and Gingeras, 2015; Krueger, 2015; de Sena Brandine and Smith, 2019).

### 2.6 Statistical analysis

Significance of the alpha diversity (mean Shannon coefficient) was evaluated starting from the Shannon coefficient of every sample using a two-sided Mann–Whitney test, since the Shapiro–Wilk test showed that not all samples were normally distributed. The significance level was chosen to be *p* < 0.05.

Experiments of virulence gene expression were performed in biological triplicates, each analyzed as technical duplicates. Significance testing was performed by a one-sided Mann–Whitney U test on a *p* = 0.05 level using the mean of the technical replicates.

To evaluate the significance of expression changes observed during transcriptome analysis, *p*-values were calculated, and Benjamini–Hochberg adjustments were performed considering a false positive rate of 10% acceptable, although no analyzed sample shows significant changes of the observed genes involved in the shikimate pathway compared with the control.

### 2.7 Data availability

Obtained raw reads of the 16 s rRNA amplicon sequencing as well as the transcriptome analysis of *Y. ruckeri* can be accessed via the NCBI database under the BioProject: PRJNA1001608.

## 3 Results and discussion

### 3.1 Effect of glyphosate, Roundup^®^, and AMPA on the gut microbiome of *Salmo trutta* f. *fario*

As the sample preparation of the gut microbiome of juvenile fish was challenging due to the small gut dimensions, previous screening and optimization of different sample preparation approaches were performed. It was not possible to extract prokaryotic DNA selectively during preparation without neglecting important compartments (i.e., mucosa-associated bacteria). The ratio of detected bacterial cell equivalents per ng of extracted DNA was an important parameter to be determined in order to assess sample quality. As the high concentration of eukaryotic DNA can negatively influence both DNA extraction and the following PCR-based molecular methods, the amount of eukaryotic DNA should be kept to a minimum. Neither the fish age nor the exposure to glyphosate, AMPA, or Roundup^®^ had a significant influence on the bacterial DNA yield of the extraction, suggesting that glyphosate, AMPA, and Roundup^®^ do not decrease the overall amount of bacteria associated with the gut. Nevertheless, the amount of DNA extracted from the older fish was, with an average of 3,729 ± 2,414 ng per gut sample, slightly higher than that of the 6-month-old fish (average 2,602 ± 904 ng DNA per gut sample). The high variance in total DNA yield is caused by the individual gut sizes and preparation variances. To visualize the amount of eukaryotic DNA, a graphical plot of the 16S rRNA copy number (as a reference for bacterial population) per ng DNA is presented in Supplementary Figure S1.

**Figure 1:**
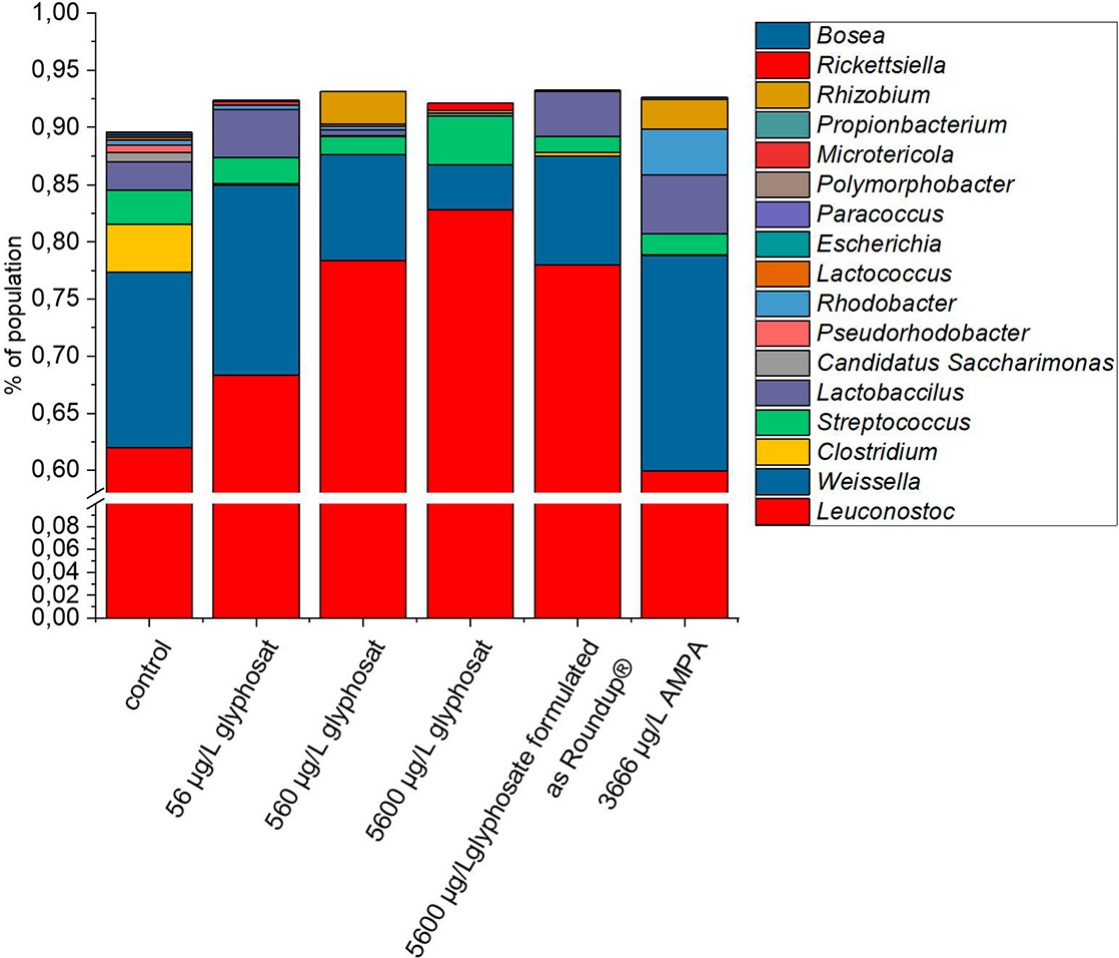
Composition of bacterial gut community from 10-month-old fish based on 16S rRNA amplicon sequencing. A number of 16 individual DNA extraction samples were qualified for high quality amplicon sequencing. For better resolution of low abundant genera, the y-axis is interrupted from 1% to 60%.

To get an overview of the microbial gut population of fish, amplicon sequencing based on 16S rRNA was performed, targeting the V1-V3 variable region. The bacterial community composition of 10-month-old fish is shown in Figure 1. Due to the DNA sample quality and amount needed for 16S rRNA amplicon sequencing, not all gut samples were suited and qualified for this analysis. Thus, the number of fish gut sample for each of the different tank treatments varied up to Δn = 4.

Each sample resulted in approximately 160,000 raw reads with 74,000 total read pairs with a mean read length of 278 bp. Depending on the sample, approximately 36,292 reads were matched. Detailed statistics for each sample are presented in Supplementary Tables S1, S2.

The control fish gut contained 17 different detectable genera. Exposure to glyphosate led to a reduction in nine different detectable bacterial genera for 56 μg L^−1^ glyphosate and seven detectable genera for 560 μg L^−1^ and 5,600 μg L^−1^ glyphosate. Exposure to Roundup^®^ led to a reduction to six detectable genera and AMPA to eight detectable bacterial genera. Commonly eliminated genera through all treatments were *Escherichia, Polymorphobacter, Paracoccus*, and *Lactococcus*. Bacterial genera, which showed resistance against Roundup^®^ and only got eliminated by higher glyphosate concentrations, were *Rhodobacter, Lactococcus, and Clostridium*. The majority of the fish gut population consisted of *Leuconostoc* (61%) followed by *Weissella* (15%). Less abundant bacteria were *Clostridium* (4%), *Streptococcus* (3%), and *Lactobacillus* (3%). It can be observed at first glance that after exposure to glyphosate, the abundance of *Leuconostoc* spp. increased approximately 22%. As *Leuconostoc* spp. is known as a probiotic bacterium, its increase would contradict the potential negative effects of glyphosate on the health of hosts (Balcázar et al., 2009; Pérez et al., 2010). A major counterpart to the increase in *Leuconostoc* spp. was the decrease in *Weissella* spp. by 11%. The remaining 11% was compensated by the decrease or even elimination of low abundant species such as *Clostridium*, *Streptococcus,* and *Pseudorhodobacter*. Some bacteria such as *Microtericola* and *Rhizobium* were only detected after exposure to one of the test substances. Overall, these bacteria-specific responses to exposure to glyphosate are caused by a wide range of susceptibilities of different bacteria species to glyphosate (Nielsen et al., 2018). It should be noted that even an increase in a bacterial genus, which is identified as probiotic, is indeed a shift of microbial population, which can lead to dysbiosis and, therefore, can have a negative effect on the health of the host.

Although a glyphosate-induced intestinal population shift could be observed, the changes in the abundance of low-abundant species might influence the vitality of hosts in a positive and a negative way, since even low abundant species in the gut microbiome have a strong impact on the vitality of hosts (Sheehan et al., 2015; Yang et al., 2019). Therefore, even the weakly observed glyphosate-induced changes in the microbiome should not be neglected.

**Figure 2:**
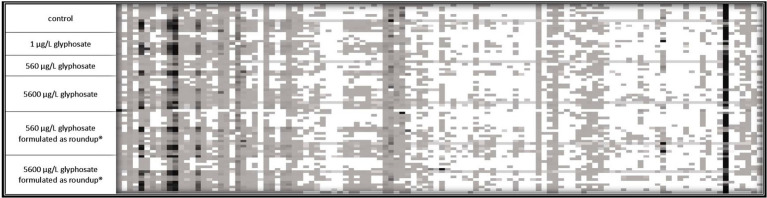
16S rDNA amplicon fragmentation pattern of the gut microbiome of 6-month-old fish. Each tile represents a phylogenetic cluster. The presence of each phylogenetic cluster is represented by the color of the tile with white as absence and deep black as predominant phylogenetic cluster. For control, 1 μg L^−1^ glyphosate and 560 μg L^−1^ glyphosate *n* = 10 for 5,600 μg L^−1^ glyphosate, 560 μg L^−1^ glyphosate formulated as Roundup^®^, and 5,600 μg L^−1^ glyphosate formulated as Roundup^®^ *n* = 15.

**Figure 3:**
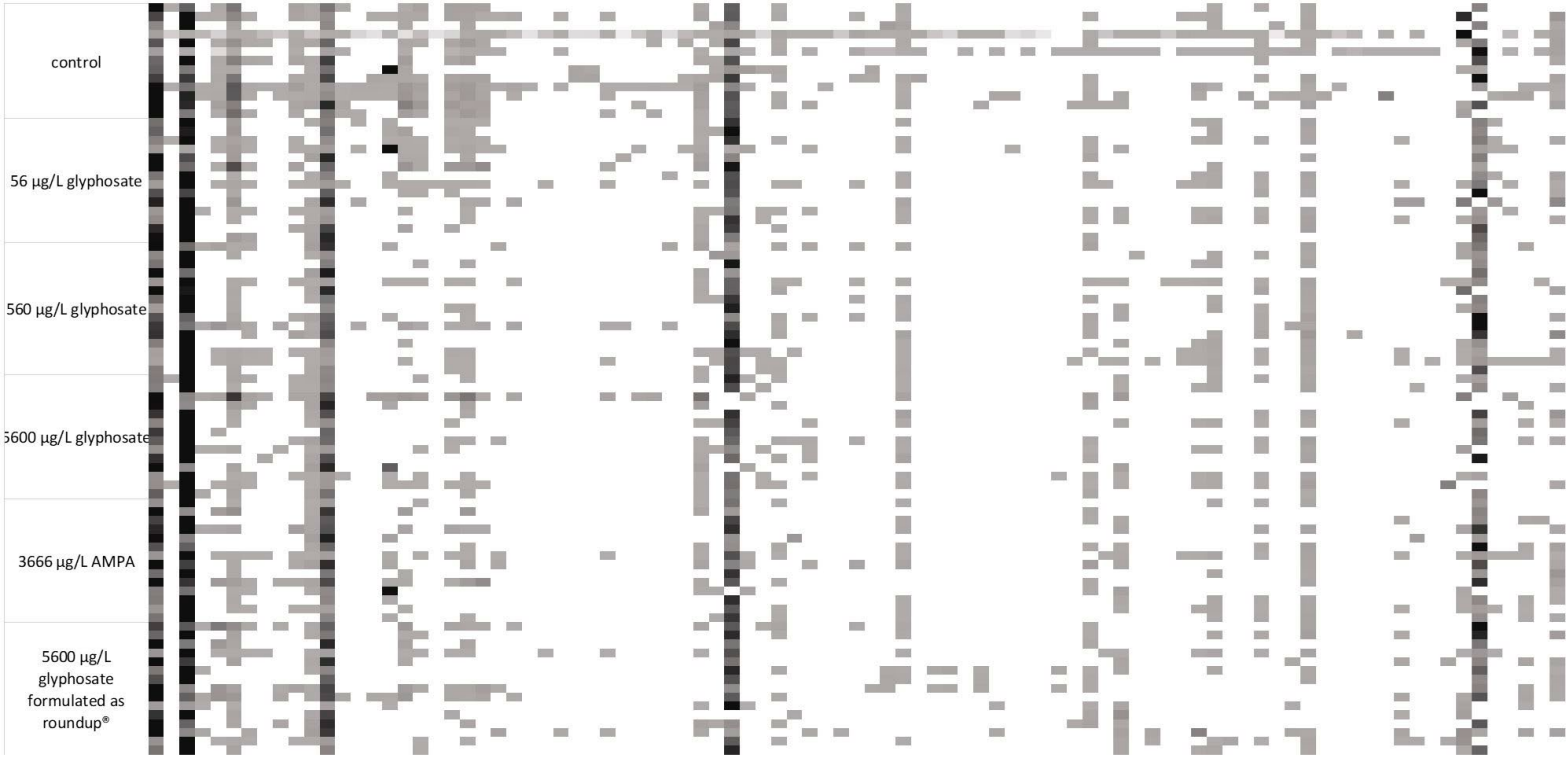
16S rDNA amplicon fragmentation pattern of the gut microbiome of 10-month-old fish. Each tile represents a phylogenetic cluster. The presence of each phylogenetic cluster is represented by the color of the tile with white as absence and deep black as predominant phylogenetic cluster. For each treatment, *n* = 15.

Fingerprint profile patterns, displaying each detected fragment obtained by the t-RFLP of both 6-month-old and 10-month-old fish, are presented in Figures 2, 3.

It became obvious by these analyses that the gut microbiome of 6-month-old fish was more diverse than the ones of 10-month-old fish. In the case of 6-month-old fish, 114 different phylogenetic clusters could be detected, while the t-RFLP of 10-month-old fish resulted in 91 different phylogenetic clusters. At both ages, dominant phylogenetic clusters, which are present in the majority of the analyzed samples and made up a majority of the population, could be observed. Additionally, some fragments were only present in some fish samples, indicating the presence of distinct bacteria in fish subpopulations. The presence of these bacteria was common for all types of fish within one tank, indicating a variance in the fish microbiome despite similar treatment. Especially for 10-month-old fish, the control fish contained some phylogenetic clusters (at the left side of the fragmentation pattern), which were absent in the majority of the gut of the exposed fish.

The decreased number of phylogenetic clusters of the microbiome of 10-month-old juvenile fish compared with 6-month-old fish leads to an assumption that the gut microbiome is altered between both age cohorts. Consequently, it was advised to analyze both age cohorts separately in order to address this diversity between the age cohorts.

### 3.2 Shannon and Bray–Curtis statistical evaluations of the t-RFLP data

The alpha diversity (Shannon coefficient) of the gut microbiome of both age cohorts is presented in Table 3. The Shannon coefficient shows the biodiversity within a single population, whereby a higher coefficient shows a higher diversity of the microbiome.

**Table 3:** Shannon coefficient of the population of 6-month-old **(A)** and 10-month-old fish **(B)** gut microbiome upon exposure to different concentrations of glyphosate, AMPA, and Roundup^®^.

A—6 months old fish	Shannon coefficient	B—10 months old fish	Shannon coefficient
Control	1.93	Control	1.82
56 μg L^−1^ glyphosate	1.95	56 μg L^−1^ glyphosate	1.64*
560 μg L^−1^ glyphosate	1.90	560 μg L^−1^ glyphosate	1.60*
5,600 μg L^−1^ glyphosate	1.98	5,600 μg L^−1^ glyphosate	1.61*
560 μg L^−1^ glyphosate formulated as Roundup^®^	1.92	3,666 μg L^−1^ AMPA	1.61*
5,600 μg L^−1^ glyphosate formulated as Roundup^®^	1.93	5,600 μg L^−1^ glyphosate formulated as Roundup^®^	1.65*

Asterisk represents a significant change in the Shannon coefficient compared with the control (*p* < 0.05).

It was obvious that, irrespective of the exposure, young fish have a more diverse gut microbiome than older fish (Shannon coefficient of 1.93 vs. 1.82). The exposure of 6-month-old fish to glyphosate, AMPA, and Roundup^®^ did not show a significant effect on the microbiome diversity. Furthermore, the concentration of glyphosate did not play any role. However, in 10-month-old fish, AMPA and Roundup^®^ affected the gut microbiome diversity in the same way as glyphosate (1.65 for 5,600 μg L^−1^ glyphosate formulated as Roundup^®^ and 1.61 for 3,666 μg L^−1^ AMPA). The control group showed a Shannon coefficient of 1.93, while the exposure groups resulted in coefficients between 1.90 and 1.98.

The Shannon coefficient gives an overview of the diversity of a microbiome within itself. Therefore, it is not suitable to elaborate on the differences between the microbiome of two different groups (microbiome dissimilarity). For this purpose, Bray–Curtis dissimilarity analysis was utilized. It allows us to directly compare the microbiomes of the untreated control group with the ones exposed to different concentrations of glyphosate, AMPA, and Roundup^®^. The resulting coefficient between 0 and 1 describes the species dissimilarity of both microbiomes, as 0 represents microbiomes with identical compositions, while a Bray–Curtis coefficient of 1 describes two microbiomes that have no common species. This allows a more direct comparison of two sets of population and gives an overview of composition changes as a result of exposure to glyphosate, AMPA, and Roundup^®^. Bray–Curtis Index of both 6-month-old and 10-month-old fish is presented in Table 4 (6-month-old fish) and Table 5 (10-month-old fish).

**Table 4:** Bray–Curtis coefficient of the gut microbiome of 6-month-old fish.

6 month old fish	Control	56 μg L^−1^ glyphosate	560 μg L^−1^ glyphosate	5,600 μg L^−1^ glyphosate	560 μg L^−1^ glyphosate formulated as Roundup^®^
Control					
56 μg L^−1^ glyphosate	0.22				
560 μg L^−1^ glyphosate	0.15	0.28			
5,600 μg L^−1^ glyphosate	0.16	0.18	0.21		
560 μg L^−1^ glyphosate formulated as Roundup^®^	0.09	0.20	0.14	0.15	
5,600 μg L^−1^ glyphosate formulated as Roundup^®^	0.15	0.18	0.22	0.13	0.14

**Table 5:** Bray–Curtis coefficient of the gut microbiome of 10-month-old fish.

10 month old fish	Control	56 μg L^−1^ glyphosate	560 μg L^−1^ glyphosate	5,600 μg L^−1^ glyphosate	3,666 μg L^−1^ AMPA
Control					
56 μg L^−1^ glyphosate	0.22				
560 μg L^−1^ glyphosate	0.28	0.12			
5,600 μg L^−1^ glyphosate	0.29	0.13	0.13		
3,666 μg L^−1^ AMPA	0.26	0.08	0.11	0.10	
5,600 μg L^−1^ glyphosate formulated as Roundup^®^	0.27	0.14	0.14	0.16	0.12

The overall impact of glyphosate, AMPA, and Roundup^®^ on the microbiome composition was evident on a small scale. The range of Bray–Curtis coefficients (beta diversity) between the control group and treated fish varied between 0.09 and 0.22 for 6-month-old fish and 0.1 and 0.29 for 10-month-old fish.

The 6-month-old fish showed the most distinct effect for the lower glyphosate concentration of 56 μg L^−1^ associated with a Bray–Curtis coefficient of 0.22. Increasing glyphosate concentration led to microbiome composition, which is again more similar to the microbiome of untreated fish. A similar hormesis effect on the microbiome of young fish could be observed in previous studies with the anti-diabetic compound metformin (Rogall et al., 2020). A concentration of 560 μg L^−1^ and 5,600 μg L^−1^ glyphosate-formulated as Roundup^®^ did not have an effect on the gut microbiome of 6-month-old fish larvae. The Bray–Curtis coefficient of the gut microbiome of 10-month-old fish increases with increasing glyphosate concentrations, starting with 0.22 for 56 μg L^−1^ glyphosate up to 0.29 for 5,600 μg L^−1^ glyphosate. Thus, glyphosate and AMPA caused comparable shift in the gut microbiome of 10-month-old fish (Bray–Curtis coefficient of 0.26 for glyphosate and 0.27 for AMPA).

Furthermore, the analyses showed that the differences between controls and treated fish were only slightly influenced by the formulation of the substance in high concentrations with a Bray–Curtis coefficient of 0.15 in 6-month-old fish and 0.26 in 10-month-old fish after exposure. However, a comparison of the Roundup©-treated fish with raw glyphosate-treated fish showed that their differences were in the same range (Bray–Curtis coefficient of 0.13 for 6-month-old fish and 0.16 for 10-month-old fish) as their comparison with the control fish, suggesting that the effect of glyphosate is not identical to the Roundup^®^ formulation containing the same amount of glyphosate.

On a final note, t-RFLP analysis verified the results of 16S rDNA amplicon sequencing while considering a wider range of fish gut microbiome samples. Population changes upon exposure to glyphosate, AMPA, and Roundup^®^ occur but are limited to a small fraction of the microbiome subpopulations. The extent of the shift does not only depend on the glyphosate concentration. The age of the fish and therein the initial composition of the microbiome and its initial diversity play an important and defining role.

### 3.3 *In vivo* detection of fish pathogens in gut microbiomes

**Figure 4:**
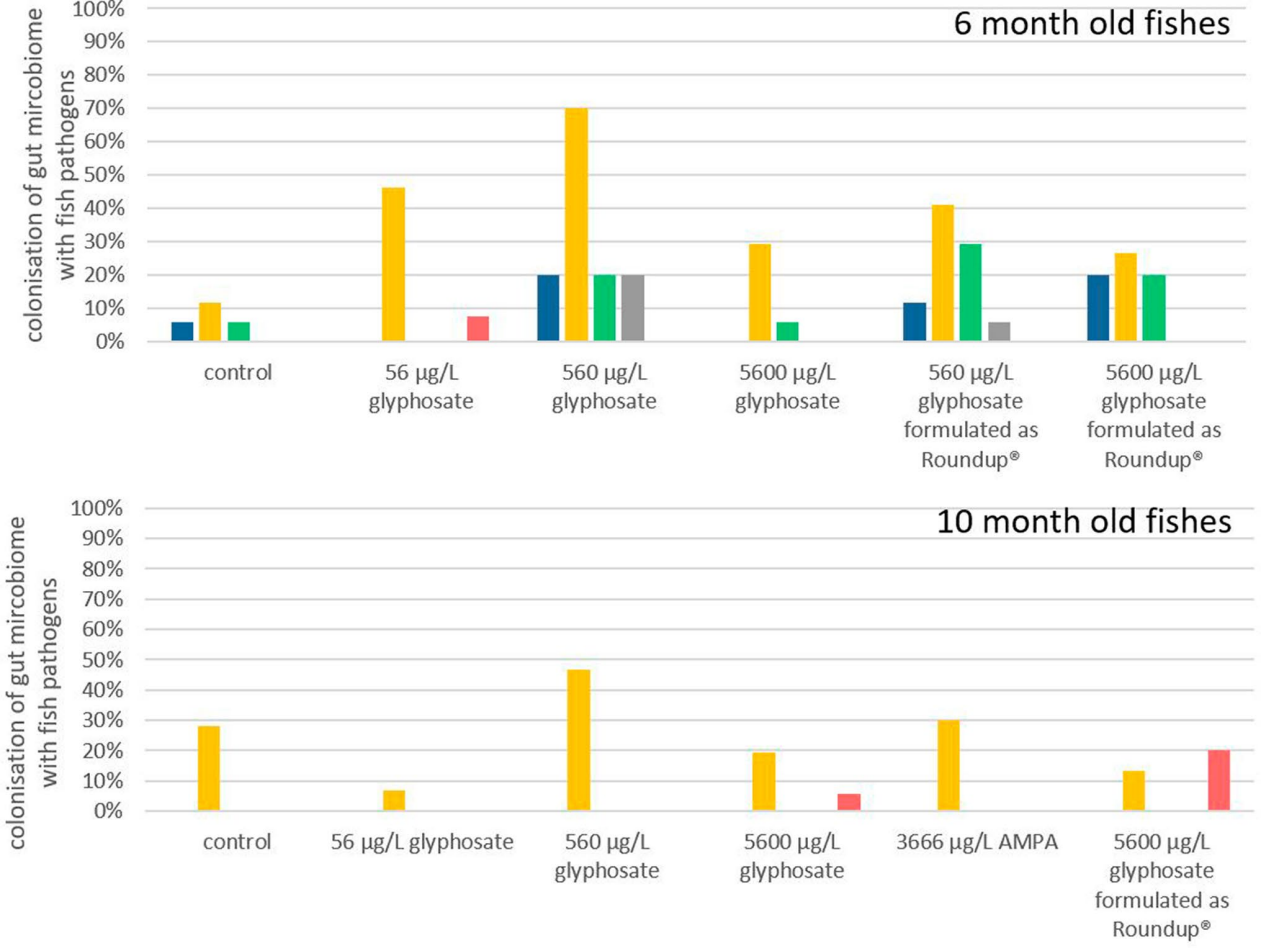
Percentage of gut microbiota which contained *A. salmonicida* (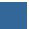), *Y. ruckeri* (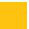), *E. faecium* (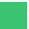), *E. faecalis* (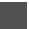), and *F. psychrophilum* (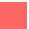) after the exposure of glyphosate and Roundup^®^. *n* = 8 to 18, depending on exposure and age.

It is hypothesized that even small changes in the microbiome can have a significant effect, especially if pathogens are involved (Sheehan et al., 2015). Each obtained gut microbiome sample was screened via qPCR for the selection of fish facultative pathogens, such as *Y. ruckeri*, *E. faecalis*, *E. faecium*, *A. salmonicida,* and *F. psychrophilum*. The frequencies in which fish microbiomes were colonized by each of these fish pathogens are presented in Figure 4. The deviation of the fish microbiome colonization rates between the different breeding tanks with identical conditions varied between 10 and 15%. It became apparent that younger fish of 6 months were more susceptible to colonization with fish pathogens than 10-month-old ones, which may also be a result of the more matured immune status and/or more stable microbiome of older fish. *Y. ruckeri* was analyzed with a percentage of colonization up to 70% being the most abundant fish pathogen. *A. salmonicida, E. faecium, E. faecalis,* and *F. psychrophilum* were less abundant as their percentage of colonization was up to 30% of all analyzed fish guts.

Exposure of 6-month-old fish to glyphosate resulted in a major rise in colonization with *Y. ruckeri*, starting from 10% for control fish to 70% for fish exposed to 560 μg L^−1^ glyphosate. The percentage of fish colonization with *A. salmonicida, E. faecium,* and *E. faecalis* did also increase to 20% upon exposure to glyphosate. It became evident that an increase in the glyphosate concentration resulted in a higher percentage of colonization up to 560 μg L^−1^ glyphosate. Rising the concentration further up to 5,600 μg L^−1^ glyphosate no longer led to an additional increase. The colonization probability is 30% lower for 5,600 μg L^−1^ glyphosate than for 560 μg L^−1^ glyphosate. Exposure to Roundup^®^ also results in an increase in *Y. ruckeri, E. faecium,* and *E. faecalis* colonization, however, not in the same way as in glyphosate-exposed fish. This became obvious, especially for *Y. ruckeri,* exhibiting a colonization percentage rise of 40% upon exposure. The colonization percentages of *A. salmonicida* and *F. psychrophilum* in 6-month-old fish were not increased significantly by any exposure of the fish.

In contrast to this, in 10-month-old fish, the percentage of colonization with pathogens was less impacted by exposure to glyphosate, AMPA, and Roundup^®^. Only *Y. ruckeri* could be frequently detected in all exposure groups. Whereas the colonization of fish guts from the control groups was at 29%, exposure to glyphosate or Roundup^®^ resulted in colonization between 5% and 45%. Thus, excluding the treatment with 560 μg L^−1^ glyphosate (with a colonization rate of 45%), no significant change in colonization with *Y. ruckeri* could be observed. High concentrations of glyphosate and Roundup^®^ led to a slight increase in colonization with *F. psychrophilum*, which could neither be detected in the fish gut of control samples nor the lower test substance concentrations. The increased colonization rate of 10-month old fish exposed to 560 μg L^−1^ glyphosate up to 45% colonization with *Y. ruckeri* goes in accordance with the colonization rates of 6-month old fish leading to a depiction of 560 μg L^−1^ to be the most effective concentration instead of an increasing effect by increasing concentrations.

As this approach and the results obtained provide the first hint of the effect of glyphosate and Roundup^®^ on the colonization of fish pathogens, it has a major drawback just like most exposure experiments: the fish grow with time and they were exposed in artificial systems under controlled environments. As the trout breeding station, where the larvae were obtained is certified as pathogen-free, it is at least questionable if the lack of colonization is due to the missing effect of exposure or the overall absence of the pathogen in the environment. This especially accounts for pathogens with low colonization rates. Due to its relatively high abundance in this setup, *Y. ruckeri* can be observed as the only reliable indicator through all exposures and ages, resulting in the final conclusion that glyphosate, AMPA, and Roundup^®^ showed an effect on the colonization of fish guts, Although the scope of the effect is, among other factors, determined by the age of the fish and the glyphosate concentration. Here, the guts of younger fish with a more diverse microbiome are more affected by pathogens than those of older fish, verifying the results obtained by t-RFLP population analysis.

#### 3.3.1 Influence of glyphosate on the transcriptome of *Yersinia ruckeri in vitro*

Since *Y. ruckeri* is an abundant fish pathogen directly identified in the fish microbiome, whole transcriptome analyses were subsequently conducted to gain a more general overview of the effect of glyphosate on the gene expression of this fish pathogen. As hypothesized, glyphosate inhibits enzymes of the shikimate pathway, resulting in the inhibition of the synthesis of aromatic amino acids. Hence, gene expression analyses under the influence of glyphosate were performed with and without amino acids present in the cultivation medium. The sequencing runs resulted in an average of 20 million reads for *Y. ruckeri* after trimming and removing ribosomal RNA. Successful mapping was possible for 97%–100% of the reads, resulting in the detection of 2,175 expressed genes for *Y. ruckeri*. A major difference in the regulation of the gene expression could be observed: *Y. ruckeri* showed a quite limited response in gene regulation when cultivated without any amino acids present in the medium (M9 medium), while when incubated after the addition of amino acids (TSB-medium), 1,661 of the 2,175 detected genes were significantly regulated when exposed to 1 μg L^−1^ glyphosate. Higher concentrations of glyphosate led to a decrease in gene regulation (total of 104 regulated genes for 560 μg L^−1^ and 3 regulated genes for 5,600 μg L^−1^ glyphosate). The regulated genes did not exhibit a trend toward upregulation or downregulation. A summary of the upregulated and downregulated gene numbers with and without the presence of amino acids is presented in Table 6.

**Table 6:** Amount of significantly regulated genes of *Y. ruckeri* upon exposure to glyphosate in the presence or absence of amino acids.

Media	Treatment	Up-regulated genes	Down-regulated genes
Without the presence of amino acids (M9-medium)	1 μg L^−1^ glyphosate	0	0
	560 μg L^−1^ glyphosate	6	10
	5,600 μg L^−1^ glyphosate	7	15
Including aromatic amino acids (TSB medium)	1 μg L^−1^ glyphosate	879	782
	560 μg L^−1^ glyphosate	64	41
	5,600 μg L^−1^ glyphosate	0	3

**Figure 5:**
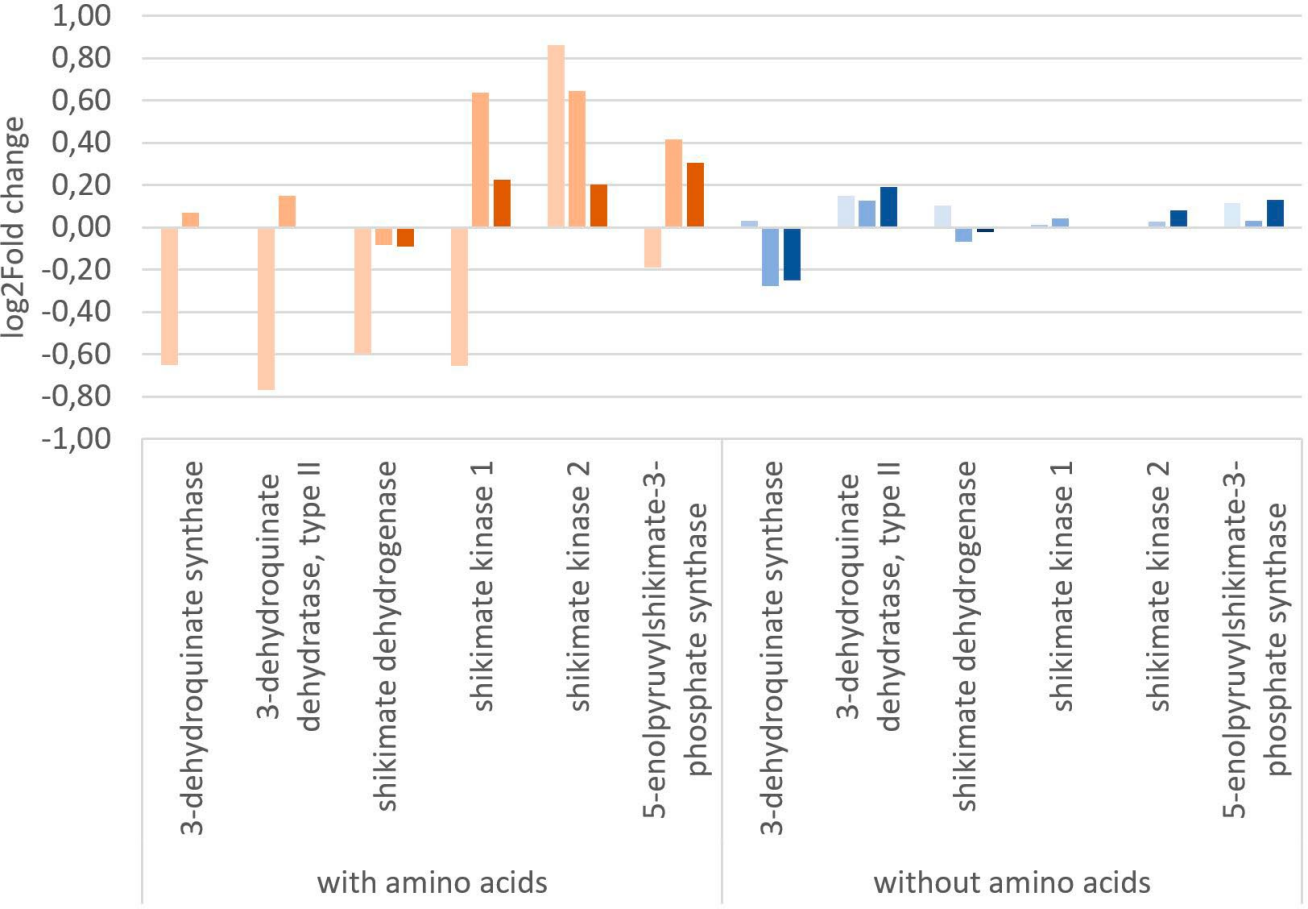
Change in gene expression of the shikimate pathway genes of *Y. ruckeri* upon exposure to glyphosate on a log_2_-fold change scale. Orange: utilizing a media containing all needed amino acids (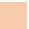: 1 μg L^−1^ glyphosate, 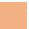: 560 μg L^−1^ glyphosate, 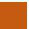: 5600 μg L^−1^ glyphosate), blue: utilizing media without any added amino acids (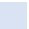: 1 μg L^−1^ glyphosate, 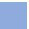: 560 μg L^−1^ glyphosate, 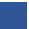: 5,600 μg L^−1^ glyphosate).

To get a more in-depth view on the effect of glyphosate on its intended target 5-enolpyruvylshikimate-3-phosphate, the expression of shikimate pathway-related genes is of special interest. Figure 5 shows the log_2_ fold change of the shikimate pathway genes of *Y. ruckeri* upon glyphosate exposure with and without the presence of amino acids. With the availability of amino acids in the medium, exposure to 1 μg L^−1^ glyphosate resulted in a decrease in the expression of five genes involved in the shikimate pathway, coding for 3-dehydroquinate synthase, 3-dehydroquinate dehydratase type II, shikimate dehydrogenase, shikimate kinase 1, and 5-enolpyruvylshikimate-3-phosphate synthase and ranging from log_2_ fold change of −0.19 to −0.77 while simultaneously the expression of shikimate kinase 2 gene increased approximately to a log_2_ fold change of 0.89. Higher concentrations of 560 μg L^−1^ and 5,600 μg L^−1^ glyphosate led to an expression increase in the genes for shikimate kinase 1 (0.6 resp. 0.2 log_2_ fold), shikimate kinase 2 (0.6 resp. 0.2 log_2_ fold), and 3-phosphoshikimate 1-carboxyvinyltransferase (0.4 resp. 0.3 log_2_ fold).

Exposure to glyphosate under circumstances without the addition of amino acids *in vivo* resulted in less expression changes up to a maximum of 0.2 log_2_ fold change. 3-Dehydroquinate synthase was expressed at lower levels with a log_2_ fold change of −0.3 upon exposure to 560 μg L^−1^ glyphosate (−0.3 log_2_ fold change for 5,600 μg L^−1^ glyphosate). The most postulated glyphosate inhibited target enzyme, i.e., 3-phosphoshikimate 1-carboxyvinyltransferases gene, however, was still expressed and also only affected up to a log_2_ fold change of maximum 0.13 at 5,600 μg L^−1^ glyphosate. No directed inhibition of this enzymatic activity could be observed in *Y. ruckeri,* a reference bacterium.

Hence, transcriptome analysis revealed that glyphosate does not have the expected impact on the gene expression in the bacterium *Y. ruckeri*. As the intended effect of glyphosate is to limit the availability of certain aromatic amino acids via the inhibition of the 5-enolpyruvylshikimate-3-phosphate (EPSP) synthase, it was expected that the gene expression of the corresponding gene increases to accommodate the low levels of aromatic amino acids and cope for the inhibition of this enzyme. However, without any additional amino acids added, no significant change in the expression of the shikimate pathway genes could be observed, hinting toward either the lack of functionality of glyphosate or, more probably, the presence of alternative pathways to bypass the need of the shikimate pathway.

In contrast to other studies that also analyzed the effect of glyphosate on the microbiomes of non-target organisms, our results showed only minor shifts in the microbial gut population. This might be due to the fact that other studies used much higher concentrations of glyphosate and AMPA or even directly fed glyphosate (Tang et al., 2020). In this context, the present study follows a more realistic approach of exposure to aquatic environments using concentrations, which are oriented to the ones observed in the environment (Busch and Reupert, 2013; LAWA – Bund/Länder-Arbeitsgemeinschaft Wasser, 2016; Carles et al., 2019). Nielsen et al. (2018) also observed only limited short-time effects of glyphosate on the microbiome upon exposure. The observed limited effect of glyphosate on real gut microbiota can be caused by at least two circumstances: (I) The effective concentration of glyphosate reaches the gut and (II) The actual need for the shikimate pathway, as complete *de novo* synthesis, is not always necessary as the c-skeletons can be reused or alternative metabolic pathways are utilized (Leibholz, 1969; Atasoglu et al., 2004; Vicini et al., 2019; Wicke et al., 2019). (III) Alteration at the catalytic center of the enzyme can lead to glyphosate-tolerant bacteria (Stalker et al., 1985). Comparable effects were also detected in artificial mixed bacteria composition, hinting to a more complex and interlinked mechanism of action (Vicini et al., 2019).

#### 3.3.2 Gene expression of bacterial virulence factors after exposure to glyphosate and AMPA *in vitro*

**Figure 6:**
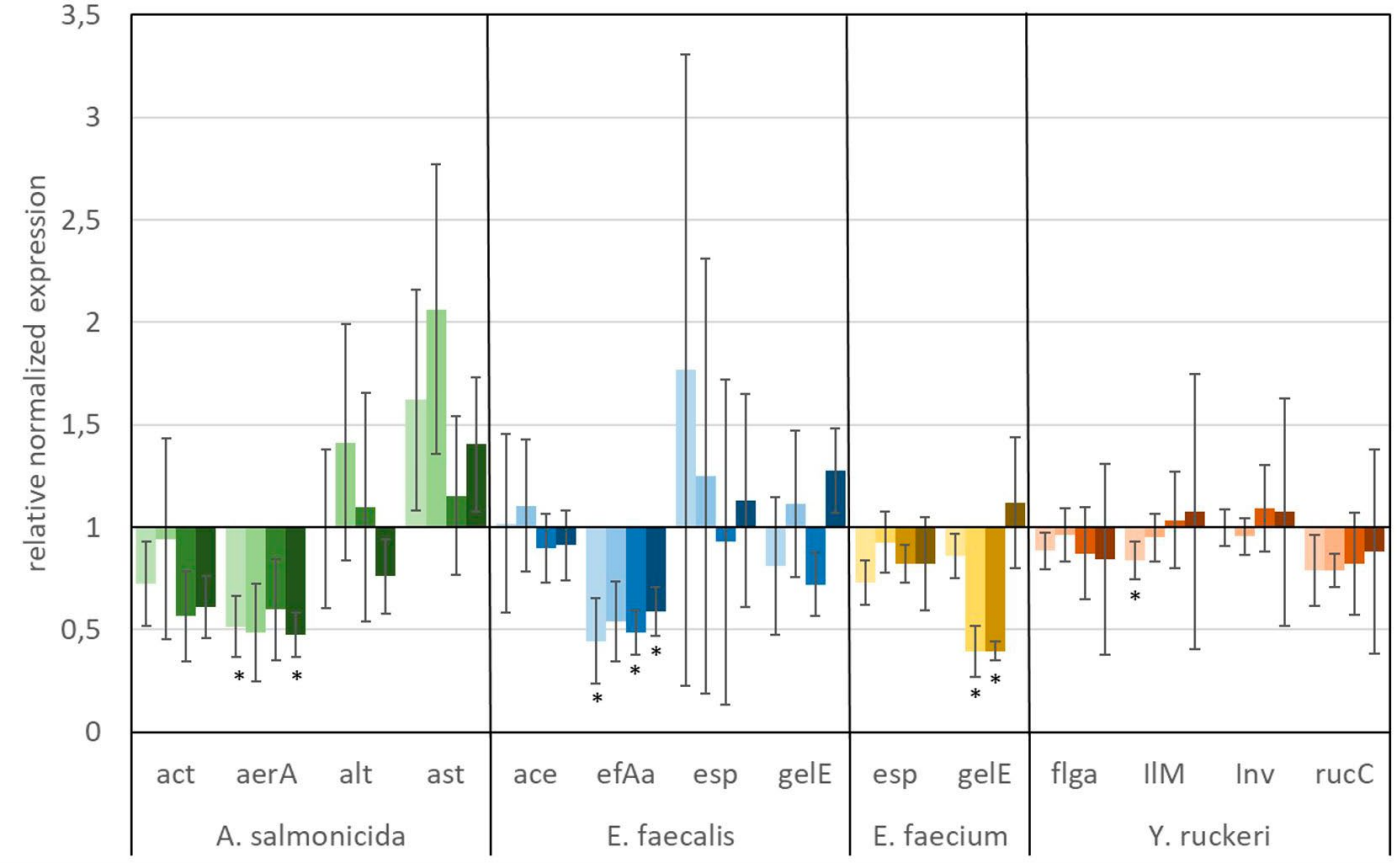
Relative normalized gene expression change of virulence factors from different fish pathogens. Significance at the *p* = 0.05 level is indicated by an asterisk. 
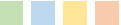
: 1 μg L^−1^ glyphosate, 
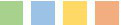
: 100 μg L^−1^ glyphosate, 
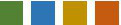
: 560 μg L^−1^ glyphosate, 
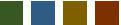
: 5,600 μg L^−1^ glyphosate. Whiskers represent standard error of the mean (SEM).

**Figure 7:**
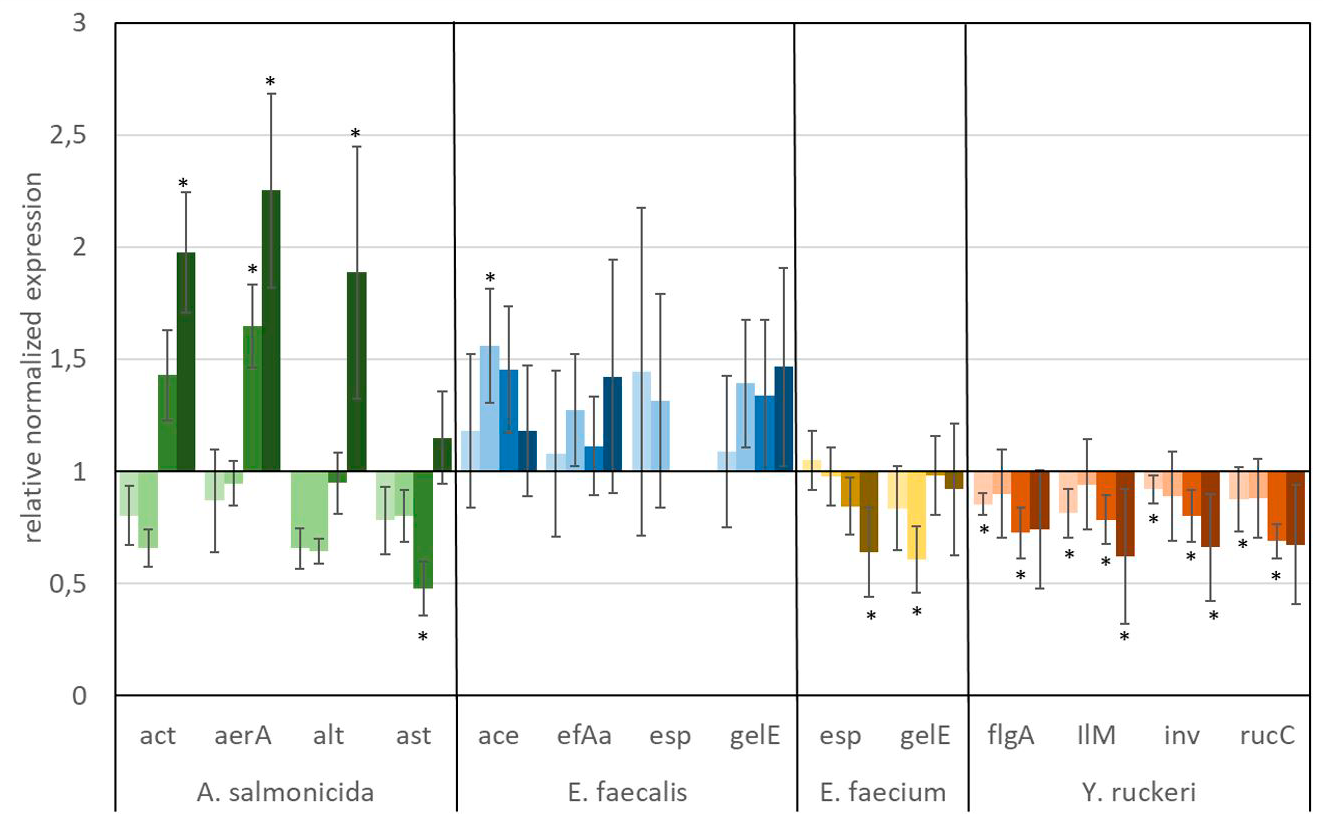
Relative normalized gene expression change of virulence factors from different fish pathogens. Significance at the *p* = 0.05 level is indicated by an asterisk. 
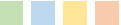
: 1 μg L^−1^ AMPA, 
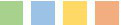
: 100 μg L^−1^ AMPA, 
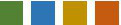
: 560 μg L^−1^ AMPA, 
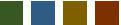
: 5,600 μg L^−1^ AMPA. Whiskers represent standard error of the mean (SEM).

The *in vitro* analyses, targeting the expression of virulence factors (see Table 2) of fish pathogens (*Y. ruckeri*, *E. faecium*, *E. faecalis*, and *A. salmonicida*) under exposure to glyphosate and AMPA, are presented in Figures 6, 7. Reference bacteria were used, and the presence of the different virulence genes was analyzed by qPCR using the gene-specific primer sets, as shown in Table 1. The gene expression analysis of virulence factors underlined their overall presence in all glyphosate and AMPA exposure experiments including the controls. The expression varied over a wide range of variability, depending on the species and the specific virulence factor. Upregulated or downregulated expression upon exposure to glyphosate or AMPA became visible.

A predominant trend of decreasing gene expression of a variety of virulence factors was observed after exposure to glyphosate (Figure 6). The expression of two virulence genes of *A. salmonicida* (*act* and *aerA*), three virulence factors of *E. faecalis* (*ace, efAa,* and *gelE*), both virulence genes from *E. faecium* (*esp and gelE*), and the four observed virulence factors of *Y. ruckeri* (*flgA, Ilm, inv, and rucC*) were negatively affected. Significant decrease in gene expression by different glyphosate concentrations was observed for the genes *aer*A of *A. salmonicida*, *efa*A of *E. faecalis*, *gel*E of *E. faecium,* and *IlM* of *Y. ruckeri*, which all exhibited downregulation of up to a 0.4-fold expression rate compared with the control. The virulence genes *alt* and *ast* of *A. salmonicida* and *esp* of *E. faecalis* showed upregulation of the gene expression up to a 2.0-fold expression rate at 560 μg L^−1^ glyphosate compared with the control. In contrast to glyphosate, exposure to AMPA (Figure 7) resulted in an overall increased virulence gene expression in *E. faecalis* (up to 1.5-fold gene expression). *A. salmonicida* showed significant (*p* = 0.01) higher gene expression after exposure to higher concentrations of AMPA (up to 2.3-fold), while the lower AMPA concentrations led to a decrease in the expression of the selected virulence factors down to 0.5-fold expression compared with the control experiment. *E. faecium* and *Y. ruckeri* exhibited an overall decreased expression of their respective virulence factors. Especially, the decreasing effect on the expression of virulence factors of *Y. ruckeri*, down to 0.7 times the control expression, was significant.

It is striking that the expression of species-specific genes, which contribute to similar tasks, was also observed to be regulated to a similar extent. The virulence factors, *aer*A and *act*, both related to aerolysin, which are correlated with diarrheal diseases, are regulated in the same way, and the same goes for the genes *esp* and *gel*E of *E. faecium,* which are involved in the biofilm formation processes (Ackermann, 2015; Morawska et al., 2022). Although identical genes were observed to be expressed differently in different species, e.g., *esp* and *gel*E of *E. faecalis* which were upregulated under exposure to AMPA, they were downregulated by *E. faecium* under identical circumstances.

The observed high variation of the gene expression, especially for single genes, upon the influence of glyphosate could be partly explained by bacteria subpopulations of the *in vitro* culture, which were distinguished by their gene expression. This so-called bet-hedging strategy was already observed in earlier studies and was a known issue in gene expression analysis (Ackermann, 2015; Morawska et al., 2022).

## 4 Conclusion

The herbicide glyphosate and its major transformation product AMPA have a distinct impact on the microbiome and its selected facultative pathogenic bacteria in brown trout (*Salmo trutta* f. *fario*). *In vivo* analysis with the gut microbiome of 6- and 10-month-old fish showed a shift of the fish microbial gut population after exposure to glyphosate, AMPA, and Roundup^®^. The extent of this population shift is highly dependent on the age of fish, wherein the gut population of 6-month-old fish was influenced to a greater extent than the microbiome of 10-month-old fish, in which the microbiome was less diverse than in the younger fish. Amplicon sequencing demonstrated that bacterial species with low abundance were more severely affected than the dominant bacterial clusters, and it has emphasized that also these low abundant bacterial species have a major effect on the viability of their hosts, especially if facultative pathogens are involved. The extent to which a decrease in bacterial diversity in the gut microbiome is or will be detrimental to fish cannot be definitively stated and requires further investigation. In this context, the observed increase in pathogen colonization after exposure to glyphosate is even more relevant for the host vitality. Considering the fact that glyphosate and AMPA induced gene expression of selective virulence factors, which are, in general, constitutively expressed in facultative pathogens, a possible impact on the vitality of the host can be discussed as a long-term effect. In addition, the specific disruption of the shikimate pathway by glyphosate, resulting in an inhibition of the synthesis of the aromatic amino acids, seems not to be the major mode of action of glyphosate in facultative pathogenic bacteria such as *Yersinia ruckeri,* which is identified in the fish microbiome. Here, the expression of enzymes of the bacterial shikimate pathway was not affected by glyphosate and AMPA. As a consequence, the decrease in the microbiome diversity and the increase in the colonization probability with fish pathogens seem to be related to the increased expression of virulence factors of pathogens, which, in the long term, can negatively influence the health of the hosts.

## Data availability statement

The datasets presented in this study can be found in online repositories. The names of the repository/repositories and accession number(s) can be found at: Bioproject accession number: PRJNA1001608.

## Ethics statement

The animal study was approved by Permission to conduct animal experiments on fish in accordance with Directive 2010/63/EU of the European Parliament and of the Council of 22 September 2010 on the protection of animals used for scientific purposes. The study was conducted in accordance with the local legislation and institutional requirements.

## Author contributions

NH: Data curation, Formal analysis, Investigation, Methodology, Visualization, Writing – original draft. VD: Investigation, Methodology, Writing – review & editing. MS: Data curation, Software, Writing – review & editing. A-KK: Software, Supervision, Writing – review & editing. H-RK: Conceptualization, Funding acquisition, Writing – review & editing. RT: Conceptualization, Funding acquisition, Project administration, Supervision, Writing – review & editing. TS: Conceptualization, Funding acquisition, Project administration, Supervision, Writing – review & editing.
