# Supplementary material for: Effect of glyphosate, its metabolite AMPA, and the glyphosate formulation Roundup® on brown trout (Salmo trutta f. fario) gut microbiome diversity
Source: Front Microbiol. 2024 Jan 15;14:1271983. doi: 10.3389/fmicb.2023.1271983 (PMC10829098; doi:10.3389/fmicb.2023.1271983)
Supplement: Supplementary file 1 [file Data_Sheet_1.docx]

Supplementary Material

Effect of glyphosate, its metabolite AMPA, and the glyphosate formulation Roundup® on brown trout (*Salmo trutta* f. *fario*) gut microbiome diversity

N. Hembach^1^, V. Drechsel^2^, M. Sobol^3^, A-K. Kaster^3^, H.-R. Köhler^2^, R. Triebskorn^2^, T. Schwartz^1*^

^1^Karlsruhe Institute of Technology (KIT), Institute of functional Interfaces, 76344 Eggenstein-Leopoldshafen, Germany

^2^University of Tuebingen, Institute of Evolution and Ecology, Animal Physiological Ecology, 72076 Tübingen, Germany

^3^Karlsruhe Institute of Technology (KIT), Institute for Biological Interfaces, 76344 Eggenstein-Leopoldshafen, Germany

*** Correspondence:** thomas.schwartz@kit.edu

# Supplementary Figures and Tables

SI table 1: 16S amplicon sequencing reads.

| ID | Total Read Pairs | Merged Read Pairs | Merging Rate | Mean Read Length (bp) |
| --- | --- | --- | --- | --- |
| 1 | 73987 | 63895 | 86.36% | 500 |
| 2 | 74243 | 66780 | 89.95% | 491 |
| 3 | 73534 | 68566 | 93.24% | 454 |
| 4 | 74115 | 65352 | 88.18% | 499 |
| 5 | 68199 | 60119 | 88.15% | 496 |
| 6 | 74095 | 66086 | 89.19% | 501 |
| 7 | 74540 | 66184 | 88.79% | 502 |
| 8 | 74201 | 67068 | 90.39% | 498 |
| 9 | 74700 | 65721 | 87.98% | 493 |
| 10 | 74097 | 67130 | 90.60% | 497 |
| 11 | 74443 | 67858 | 91.15% | 495 |
| 12 | 74489 | 66513 | 89.29% | 504 |
| 13 | 74782 | 67297 | 89.99% | 502 |
| 14 | 74461 | 66842 | 89.77% | 500 |
| 15 | 73978 | 65650 | 88.74% | 504 |
| 16 | 74315 | 67564 | 90.92% | 492 |

SI table 2: Mapping key-values of 16S amplicon sequencing.

| Sample | Input sequences | Sequences after preprocessing | Sequences after chimera removal | Sequences assigned to OTUs | Sequences assigned to taxa | Median sequence length after preprocessing |
| --- | --- | --- | --- | --- | --- | --- |
| 1 | 73776 | 73718 | 73438 | 32516 | 32516 | 497 |
| 2 | 74235 | 74212 | 73743 | 35411 | 35411 | 497 |
| 3 | 73518 | 73496 | 72973 | 40297 | 40297 | 437 |
| 4 | 73778 | 73708 | 73497 | 34309 | 34309 | 497 |
| 5 | 67112 | 66825 | 66662 | 33133 | 33133 | 497 |
| 6 | 73790 | 73713 | 73492 | 36514 | 36514 | 497 |
| 7 | 74311 | 74255 | 74154 | 36585 | 36585 | 497 |
| 8 | 74185 | 74167 | 73874 | 34797 | 34797 | 497 |
| 9 | 73701 | 73476 | 73212 | 37490 | 37490 | 497 |
| 10 | 74072 | 74050 | 73621 | 36717 | 36717 | 497 |
| 11 | 73920 | 73776 | 73473 | 39416 | 39416 | 497 |
| 12 | 74474 | 74453 | 74203 | 34760 | 34760 | 497 |
| 13 | 74775 | 74758 | 74495 | 36222 | 36222 | 497 |
| 14 | 74455 | 74427 | 74122 | 38231 | 38231 | 497 |
| 15 | 73956 | 73934 | 73559 | 36512 | 36512 | 497 |
| 16 | 74236 | 74190 | 73625 | 37777 | 37777 | 497 |
| TOTAL | 1178294 | 1177158 | 1172143 | 580687 | 580687 | 493 |

## Supplementary Figures

SI figure 1: Bacterial gene copies in 1 ng of DNA based on 16S rDNA for both 10 and 6 month old fish. Median is represented by the black bar and the mean by the small rectangle. The box shows the upper and lower quantiles, whiskers represent the minimum and maximum value.
